# New State Medical Society

**Published:** 1906-06

**Authors:** 


### NEW STATE MEDICAL SOCIETY.

BELOW we give an account, as it appeared in the *Atlanta Constitution,* of the movement to establish a new medical association in the State. The views and purposes of the organizers are set forth in the interview of Dr. Stewart:

A new State Medical Association is now being formed through the efforts of a number of the leading physicians of Georgia, and will shortly be launched with a large membership.

Those promoting the new association insist that in organizing it they are in no sense making a fight on the present Georgia Medical Association, which two years ago was amalgamated with the American Medical Association, but that they simply object to some features of the national association and are forming a body in which they will be free to act for themselves. No one will be excluded on account of membership in any other association, but on the other hand, those interested in the new association hope that many members of the old association will also become members of the new body.

It is declared that the new association will have many valuable features which have never before been introduced by a medical society in this section, and through it it is hoped that much will be done for the advancement of medical science in Georgia, and consequently for the general good.

Great interest is being shown in the movement all over Georgia, and at a preliminary meeting to be held in Atlanta at an early date leading physicians from every part of the State will be present. For several days a number of prominent physicians have been in consultation in Atlanta, and as a result, a circular letter is being sent out to the doctors of the State in the interest of the new organization.

Dr. W. W. Stewart, of Columbus, a member of the State Board of Health, who is taking a prominent part in the organization of the new association, when seen by a representative of the *Constitution,* at first refused to make any statement at all, but after being shown that the *Constitution* was in full possession of the facts as to the movement, and being assured that publication was going to be made, whether or not official authorization was given, said:

"Yes, it is true that we are organizing a new medical association; in fact, it is practically organized. We intend to make the new association a great force in Georgia, but it must be distinctly understood that we are not making a fight on the Georgia Medical Association. Some of us are opposed to the amalgamation of the old society to which we were greatly attached with the American Medical Association, and did not care to put ourselves under the restrictions required by membership in that body. Our association will be open to all reputable physicians, and we trust that many of those in the Georgia Medical Association will also unite with our association, which is to have many new features which we are confident will be of great value to the profession in Georgia.

"I would like to say in regard to this association, that its organization is not in the slightest degree retaliative toward the present Georgia Medical Association, as it will have quite a number of peculiar features never before incorporated in the plan of action of any medical association, making it unique amongst all others. To become enrolled in its membership will be as advantageous to those now in the old association as to those who are forming the new society."

The circular letter sent out is in part as follows:

"We believe this call will in every part of the State be received by the doctors as glad tidings of joy, as it means the organization of a State Medical Association hampered in no way by national influence and in every way an old high-standard Georgia Medical Association for the furtherance of scientific research and the infusion into its members of high ethical principles with brotherly love, giving every right to all and no special privilege to any.

"With your valuable cooperation in this movement we feel that to organize such an association is a matter of the greatest ease. It is not the purpose of this association to exclude from membership men belonging to any other association or scientific organization. The only requirement for organization therein will be that he be a properly graduated physician from some reputable institution recognized as being a regular school in good repute, indorsed only to the extent required by our old Georgia Association, membership in local organizations being a matter left entirely to himself."

